# Trisferrocenyltrithiophosphite-Copper(I) Bromide Composites for Electrochemical CO_2_ Reduction

**DOI:** 10.3390/ijms27020789

**Published:** 2026-01-13

**Authors:** Mikhail Khrizanforov, Ilya Bezkishko, Anastasiia Samorodnova, Ruslan Shekurov, Radis Gainullin, Kirill Kholin, Igor Yanilkin, Aidar Gubaidullin, Alexey Galushko, Vasili Miluykov

**Affiliations:** 1 Zelinsky Institute of Organic Chemistry, Russian Academy of Sciences, Leninsky Prospekt 47, 119991 Moscow, Russia; apsamorodnova@gmail.com (A.S.);; 2 Arbuzov Institute of Organic and Physical Chemistry, FRC Kazan Scientific Center, Russian Academy of Sciences, 8 Arbuzov Str., 420088 Kazan, Russia; bezkishko@gmail.com (I.B.); aidar@iopc.ru (A.G.); miluykov@iopc.ru (V.M.); 3 Department of Physics, Kazan National Research Technological University, 68 Karl Marx Str., 420015 Kazan, Russia; radis.g@mail.ru (R.G.); kholin06@mail.ru (K.K.); 4 Institute of Physics, Kazan Federal University, 18 Kremlyovskaya Str., 420008 Kazan, Russia

**Keywords:** CO_2_RR, copper(I) complexes, ferrocene derivatives, trithiophosphite ligands, electrocatalysis

## Abstract

Copper-based catalysts have emerged as promising materials for electrochemical carbon dioxide reduction reactions, owing to copper’s unique ability to facilitate multi-electron transfer processes and produce valuable products such as methanol and ethanol. In this study, novel trisferrocenyltrithiophosphite–copper(I) bromide composites with Cu-to-ligand molar ratios of 1:1 and 2:1 were synthesized and evaluated for their catalytic performance. The composites were characterized by a combination of techniques, including powder X-ray diffraction (PXRD), linear sweep voltammetry (LSV), potentiostatic testing, chromatographic analysis, scanning electron microscopy (SEM) and X-ray photoelectron spectroscopy (XPS). Electrochemical measurements demonstrated significant current enhancements in the presence of CO_2_, highlighting the composites’ catalytic activity. Potentiostatic tests revealed excellent stability, with only a 9% decline in current density over 5 h of electrolysis. Product analysis via gas chromatography indicated the formation of methanol for the 1:1 composite and ethanol for the 2:1 composite with Faradaic efficiencies of 5.79% and 9.26%, respectively. While absolute efficiencies remain modest due to competitive hydrogen evolution, these results demonstrate a tunable catalytic performance based on the Cu-to-ligand ratio. SEM and XPS studies further supported the formation of active catalytic centers and changes in the oxidation states of copper during CO_2_ reduction. PXRD analysis confirmed the retention of structural integrity for both composites before and after catalytic testing.

## 1. Introduction

The electrochemical reduction of carbon dioxide (CO_2_RR) is an attractive strategy for converting an abundant and inexpensive carbon feedstock into value-added fuels and chemicals while storing renewable electricity. Among the many possible products, liquid oxygenates are of particular interest because of their higher energy density and ease of handling. However, selective CO_2_ conversion to alcohols, especially multicarbon alcohols, remains challenging due to complex multi-electron/proton transfer pathways and competitive hydrogen evolution. Copper-based catalysts are especially promising for CO_2_RR because copper uniquely enables the formation of both hydrocarbons and alcohols, including methane, ethylene, methanol, and ethanol [1]. Nevertheless, high overpotentials and insufficient control over product distribution still limit practical applications. In particular, the selective formation of ethanol and other C_2+_ oxygenates typically requires a catalyst architecture that can promote C–C coupling while stabilizing reactive intermediates. Accumulating evidence shows that the structural features of copper catalysts strongly influence product selectivity. Specific crystallographic facets, such as Cu(100) and high-index surfaces, favor C–C interactions of adsorbed *CO species and thus promote C_2_ product formation [2,3,4,5,6,7,8,9,10,11,12].

In addition, oxidation state effects and interfacial architectures can be crucial for C_1_ alcohols. For example, a MOF-derived Cu@Cu_2_O system featuring Cu(0)/Cu(I) interfaces achieved a peak Faradaic efficiency for methanol of about 45% at −0.7 V vs. RHE, highlighting the importance of synergistic Cu(0)/Cu(I) sites for selective hydrogenation pathways. For ethanol formation, the proximity of copper centers appears to be a key prerequisite. Isolated Cu sites are generally insufficient for efficient C–C coupling, whereas adjacent Cu centers can act cooperatively [13]. A striking example is the dynamic transformation of atomically dispersed Cu into small Cu_n_ clusters under operating potentials, which enabled highly selective CO_2_-to-ethanol conversion with Faradaic efficiencies approaching ~91% under optimized conditions [14].

Similarly, ultrahigh-density Cu–N_3_ single-atom motifs on carbon supports can function as closely spaced neighboring sites, delivering ethanol FE of ~81.9% with notable stability in an H-cell configuration [14].

These studies collectively emphasize that controlling the local arrangement of Cu centers is a powerful route to steer CO_2_RR toward C_2_ oxygenates.

Alongside copper systems, the broader CO_2_RR field continues to advance rapidly. Recent reports on bismuth-based electrocatalysts, such as Bi_2_/BiVO_4−x_ heterostructures and VOx-mediated Bi–Sn alloys, have achieved very high selectivity for formate over wide potential windows, providing important benchmarks for modern CO_2_RR catalyst design [15,16].

Although these systems target different products, they underscore how rational control of active-site environment can dramatically enhance selectivity. An alternative and complementary way to control CO_2_RR selectivity is to employ molecular or hybrid catalysts in which the coordination environment is defined by the ligand framework [17]. Macrocyclic systems and other ligand-controlled architectures can enable multi-electron pathways; for instance, cobalt phthalocyanine supported on carbon materials has been reported as an efficient route toward methanol under appropriate conditions [18].

In this context, phosphorus–sulfur ligands are particularly attractive because of their strong affinity toward soft metal centers and their tunable electronic properties [19]. Incorporation of redox-active fragments, such as ferrocene, may further assist electron-transfer steps and enable additional control over catalytic behavior.

In this work, we report trisferrocenyltrithiophosphite–copper(I) bromide composites as tunable electrocatalysts for CO_2_ reduction. By varying the CuBr-to-ligand ratio under otherwise identical conditions, the product distribution can be shifted between C_1_ and C_2_ alcohols: a 1:1 composite preferentially yields methanol, whereas an increased copper content (2:1) promotes ethanol formation. This stoichiometry-controlled approach offers a simple and conceptually distinct handle to modulate CO_2_RR selectivity in a hybrid copper–ligand system, bridging features of molecular coordination control with the reactivity patterns of copper-based electrocatalysis.

## 2. Results and Discussion

Copper(I) bromide (CuBr) was mixed with triferrocenyltrithiophosphite [20] (P(SFc)_3_) in molar ratios of 1:1 and 2:1 and CH_3_CN was added. Further, the reaction mixture was stirred at room temperature for 20 h, then solvent was removed in vacuum and the product was extracted with toluene. Toluene solution was filtered and evaporated to dryness to give light brown and brown powder. Each mixture was combined with a pre-prepared carbon paste, which was formulated using carbon powder and a phosphonium-based ionic liquid [21], to give corresponding copper–trisferrocenyltrithiophosphite composite systems **1** and **2**. The ionic liquid component, known for its excellent conductivity and stability, has been previously validated as an effective medium for studying both electrochemical and electrocatalytic properties.

**Figure 1:**
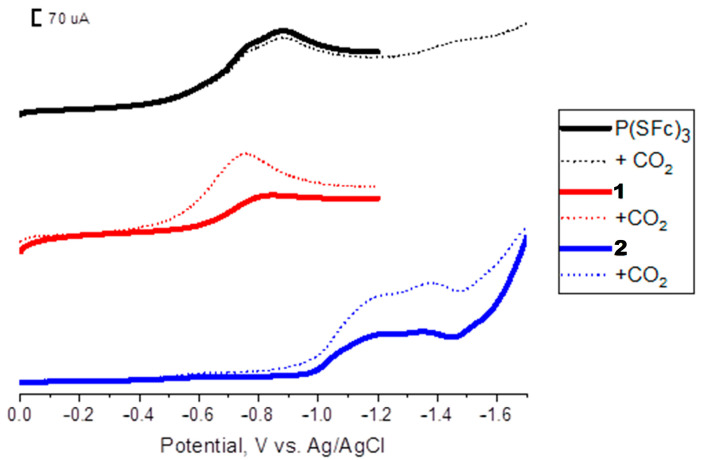
LSV profiles of composites doped with P(SFc)_3_ (black), the 1:1 CuBr-P(SFc)_3_ composite (**1**) (red), and the 2:1 CuBr-P(SFc)_3_ composite (**2**) (blue) in the presence (dots) and absence (lines) of CO_2_ in a 0.1 M KHCO_3_ solution.

The electrochemical behavior of the prepared composites **1** and **2** was investigated and compared to the individual components (CuBr and (P(SFc)_3_). Interestingly, the linear sweep voltammetry (LSV) profiles of the composites demonstrated distinct differences compared to those of the individual components, as shown in Figure 1. While CuBr and P(SFc)_3_ exhibit their characteristic electrochemical features independently, the composites show significant modifications in their redox behavior under a CO_2_ atmosphere. This increase in current density indicates CO_2_-dependent catalytic activity. Notably, this phenomenon was absent in the LSV profiles of pristine CuBr and P(SFc)_3_ when tested individually under identical conditions, highlighting the synergistic effect of combining both components into a composite material.

Figure 1 shows LSV profiles of P(SFc)_3_-doped carbon paste (black), composite **1**(red), and composite **2** (blue) in 0.1 M KHCO_3_ solution recorded under N_2_ and CO_2_. For the doped P(SFc)_3_ system, no significant increase in current was observed upon CO_2_ introduction, consistent with the lack of catalytic activity for CO_2_ reduction by P(SFc)_3_ alone. In contrast, both the **1** (1:1) and **2** (2:1) composites exhibited notable current enhancements upon CO_2_ bubbling, indicating active participation in CO_2_ electroreduction processes. The enhanced current response under CO_2_ suggests that integrating CuBr with P(SFc)_3_ creates a new catalytic environment that facilitates electron transfer and activation of CO_2_.

**Figure 2:**
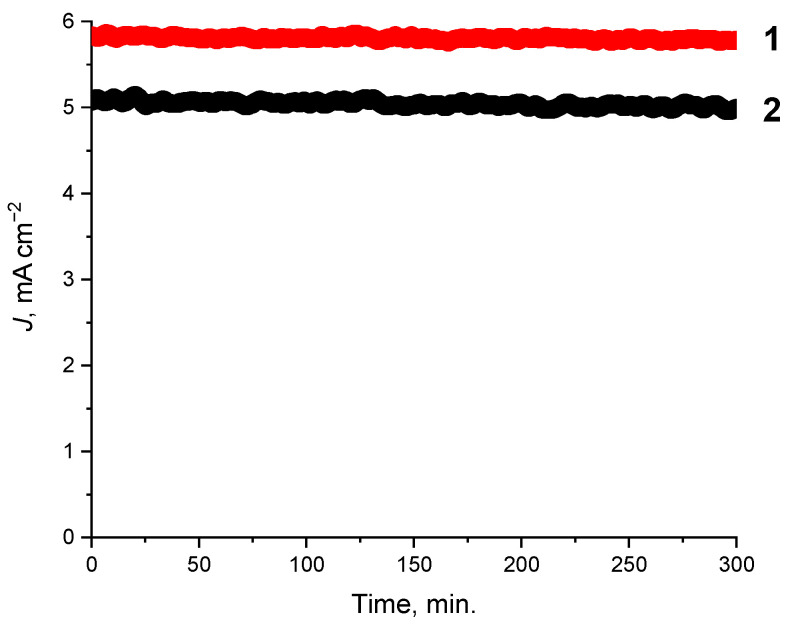
Potentiostatic tests of the composites doped with the 1:1 and 2:1 CuBr-P(SFc)_3_ composites **1** (−0.8 V vs. Ag/AgCl) and **2** (−1.2 V vs. Ag/AgCl), showing current density over 5 h of electrolysis in a 0.1 M KHCO_3_ solution under CO_2_ flow.

To evaluate the long-term stability and performance of the synthesized composites in CO_2_ reduction reactions, potentiostatic electrolysis was conducted in a 0.1 M KHCO_3_ solution under a continuous flow of CO_2_. The results demonstrate good stability and activity for the composites. During the 5 h electrolysis, the current density exhibited only a minor decline of approximately 9%, as shown in Figure 2. This performance demonstrates the robustness of the CuBr-P(SFc)_3_ composites (**1, 2**) and their ability to maintain catalytic activity under continuous CO_2_ electrolysis. The synergistic interaction between the metal center and the phosphorus-sulfur ligand framework likely contributes to the observed stability by maintaining the active sites structural integrity throughout the reaction. These results position the synthesized composites **1** and **2** as highly promising materials for scalable and sustainable applications in CO_2_RR.

**Figure 3:**
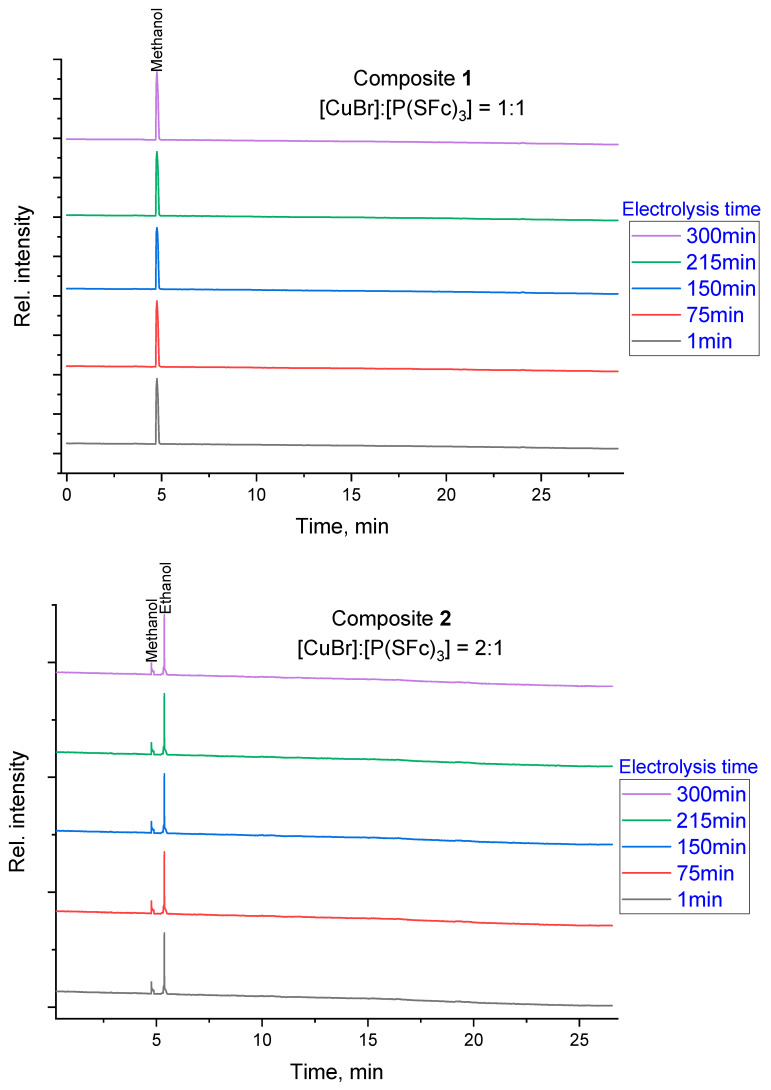
GC-FID chromatograms recorded during electrolysis using a PEG column. The time-dependent product distribution is shown for composite **1** (1:1 ratio) and composite **2** (2:1 ratio) at intervals of 1, 75, 150, 215, and 300 min. The y-axis ‘Relative Intensity’ represents normalized detector response with traces vertically offset to illustrate the consistency of product identification over time.

Chromatographic analysis was performed to elucidate the product distribution resulting from the electrochemical reduction of CO_2_ using composites **1** and **2**. The analysis revealed that the product composition depends significantly on the chosen metal-to-ligand ratio. As shown in Figure 3, each chromatographic trace was normalized and vertically offset to highlight the characteristic peaks for methanol and ethanol across different electrolysis times. This qualitative visualization confirms that the catalytic system maintains its selectivity for specific alcohols throughout the duration of the test, with composite **1** favoring methanol and composite **2** favoring ethanol. Quantitative determination of product amounts was conducted using external calibration and integrated peak areas to calculate Faradaic efficiencies.

For the composite **1**, prepared with a 1:1 molar ratio of CuBr to P(SFc)_3_, selective formation of methanol as the primary product was observed in the chromatograms. In contrast, the composite **2**, prepared with a 2:1 CuBr-to-P(SFc)_3_ ratio, demonstrates the formation of ethanol as the predominant product, while methanol remained a minor product. This shift in product profile suggests that the increased copper content in the 2:1 composite introduces additional active sites or alters the reaction mechanism, thereby enabling the formation of a broader range of reduction products. Based on quantitative GC-FID analysis and the total charge passed during potentiostatic CO_2_ electrolysis, the Faradaic efficiencies for liquid alcohols were determined. Using a total charge of Q = 50 C, methanol formation corresponds to an FE of 5.79% (5 µmol), whereas ethanol formation corresponds to an FE of 9.26% (4 µmol). The remaining charge is attributed mainly to the competing hydrogen evolution reaction (HER), which frequently dominates copper-based CO_2_RR in aqueous bicarbonate electrolytes [22].

Following electrolysis, the surfaces of the prepared composites **1** and **2** were analyzed using scanning electron microscopy (SEM) and elemental mapping, to examine the morphological and compositional changes induced by catalytic activity. SEM images revealed the formation of large, well-defined active centers on the composite surfaces, which are likely responsible for the high catalytic activity observed during CO_2_ reduction. Elemental mapping further confirmed the homogeneous distribution of key elements, including copper, phosphorus and sulfur, across the composite surfaces. This uniformity suggests effective integration of the trisferrocenyltrithiophosphite ligand and copper(I) bromide into the composite matrix, ensuring consistent active site formation.

**Figure 4:**
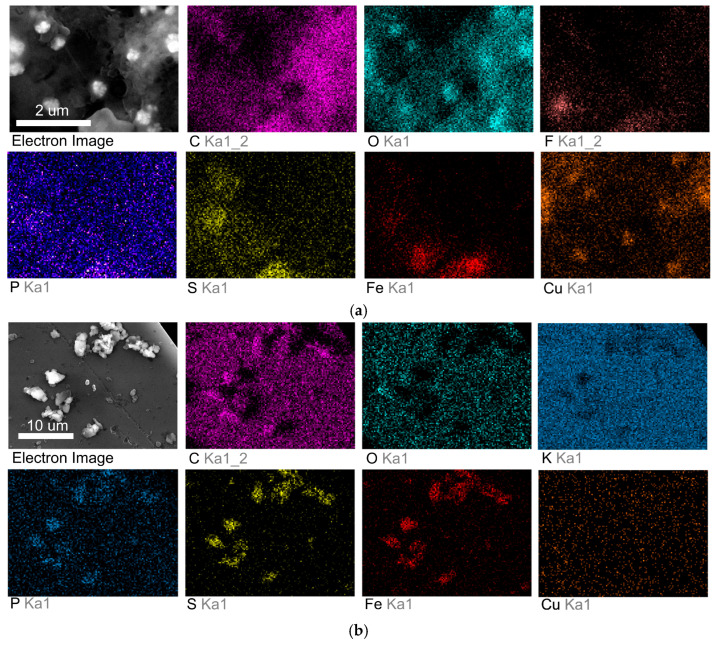
(**a**) SEM image and elemental mapping of the electrode surface for the 2:1 CuBr–P(SFc)_3_ composite **2** after CO_2_ electrolysis. (**b**) SEM image and elemental mapping of the electrode surface for the 1:1 CuBr–P(SFc)_3_ composite **1** after CO_2_ electrolysis.

The elemental analysis for the 2:1 CuBr–P(SFc)_3_ composite **2** under CO2 flow (Figure 4a) demonstrated significant copper enrichment, indicating that the catalytic process involves active participation of the copper sites. Similarly, the 1:1 CuBr–P(SFc)_3_ composite **1** (Figure 4b) displayed a high density of copper and phosphorus elements. Semi-quantitative EDX mapping results were consistent with the nominal Cu:ligand ratios of the prepared composites **1** and **2**.

**Figure 5:**
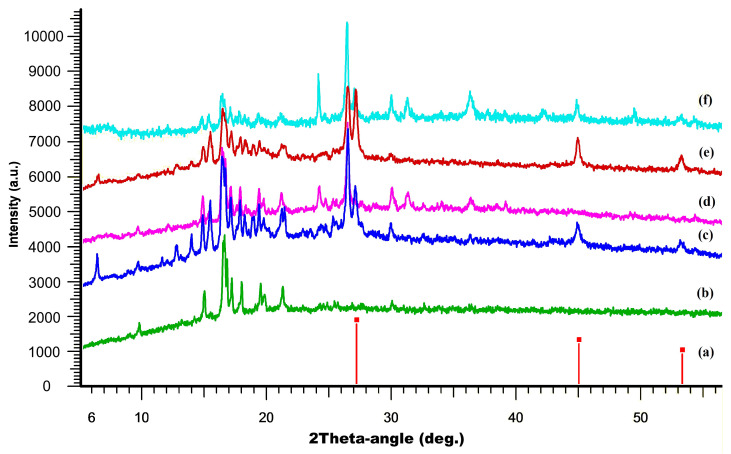
PXRD patterns (from bottom to top): of CuBr from PDF-2, Ref. code 00-001-0683, vertical red lines (a), experimental curve of P(SFc)_3_ (green curve) (b), the composite prepared with a 1:1 molar ratio of CuBr to (P(SFc)3 (blue curve) (c), the composite prepared with a 1:1 molar ratio of CuBr to (P(SFc)_3_ after electrocatalysis (magenta curve) (d), the composite prepared with a 2:1 molar ratio of CuBr to (P(SFc)_3_ (brown curve) (e) and the composite prepared with a 2:1 molar ratio of CuBr to (P(SFc)_3_ after electrocatalysis (light blue curve) (f).

To confirm the structural integrity and composition of the synthesized composites, powder X-ray diffraction (PXRD) analysis was performed (Figure 5). The PXRD patterns of the composites were compared to those of the individual components, namely CuBr and (P(SFc)_3_. The results demonstrated that the characteristic diffraction peaks associated with both CuBr and P(SFc)_3_ were retained in the composites. It is obvious that during the synthesis process the initial components are also preserved in their individual, undamaged form. However, the presence of a significant number of other intense diffraction peaks indicates a multicomponent composition of both samples and the presence of additional crystalline phases, suggesting additional crystalline phases, possibly arising from Cu–ligand coordination. After the potentiostatic tests, PXRD analysis revealed that the composite with Cu-to-ligand ratios of 2:1 (CuBr and P(SFc)_3_) largely retains its initial diffraction features, but additional peaks appear, indicating the possible formation of additional crystalline phases. Decreasing the Cu:ligand ratio to 1:1 (CuBr and P(SFc)_3_) in another composite led to the disappearance of the peaks of the individual crystalline copper bromide, while generally maintaining the rest of the component composition. This suggests that the majority of the composite maintains its structural integrity under catalytic conditions.

XPS analysis was conducted to provide insights into the oxidation states of copper, iron, and phosphorus, as well as the surface composition of the prepared composites before and after electrochemical reduction of CO_2_. The results demonstrate significant changes in the electronic environment of the key elements, reflecting the role of the composites in CO_2_ electroreduction.

**Figure 6:**
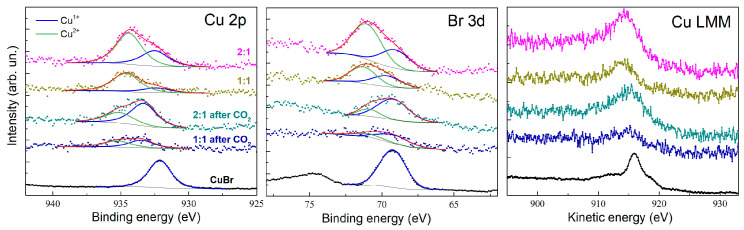
XPS spectra of Cu 2p_3/2_, Br 3d, and Cu LMM for CuBr and CuBr–(P(SFc)_3_ composites with molar ratios of 1:1 and 2:1, before and after CO_2_ electrolysis. The spectra highlight the coexistence and changes in Cu^+^ and Cu^2+^ species as well as bromide ion retention during catalytic processes.

Cu^+^ and Cu^2+^ species in the pristine composites with molar ratios of 1:1 and 2:1 (CuBr-to-(P(SFc)_3_) [23]. The relative intensity of the Cu^+^ signal dominates in both cases, indicative of the stabilization of Cu(I) by the ligand environment. After CO_2_ electrolysis, the Cu^+^ content significantly increases, particularly in the 2:1 composite, as evidenced by the reduced Cu^2+^. This observation suggests the active participation of Cu(I) centers in the catalytic cycle, with Cu(II) likely serving as a transient intermediate during electron transfer. The Br 3d spectra (Figure 6, center) show clear peaks corresponding to bromide ions in the CuBr precursor and composites. The intensity of these peaks decreases after electrolysis, suggesting partial displacement of bromide ions during catalytic activity, possibly due to the interaction with CO_2_ or intermediates.

**Figure 7:**
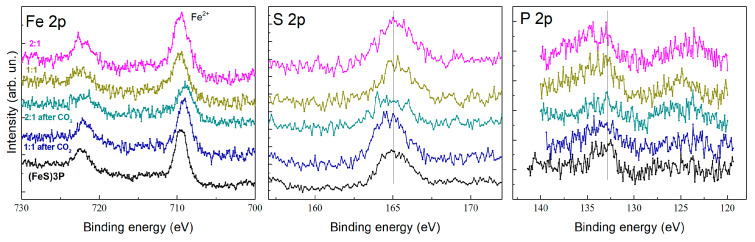
XPS spectra of Fe 2p, S 2p, and P 2p for (P(SFc)_3_, CuBr–(P(SFc)_3_ composites with molar ratios of 1:1 and 2:1, before and after CO_2_ electrolysis.

The Fe 2p spectra (Figure 7, left) confirm the presence of Fe^2+^ in the composites, consistent with the triferrocenyl structure of the (P(SFc)_3_ ligand. After electrolysis, changes in the Fe 2p binding energy suggest slight modifications in the electronic environment of the ferrocene units, potentially due to ligand reorganization or interaction with CO_2_ intermediates. The S 2p spectra (Figure 7, center) indicate the retention of sulfur’s binding environment, supporting the stability of the trithiophosphite ligand under electrochemical conditions. The P 2p spectra (Figure 7, right) exhibit peaks consistent with phosphorus bound to sulfur in the trithiophosphite ligand. After electrolysis, subtle shifts in the P 2p binding energy suggest minor alterations in the ligand’s electronic structure, likely due to interactions with copper centers or CO_2_ during catalysis.

The increased proportion of Cu^+^ after CO_2_ electrolysis highlights its role as the primary active species in the catalytic process. Changes in the Fe 2p and P 2p spectra suggest that the ligand framework actively participates in stabilizing catalytic intermediates and facilitating electron transfer. Bromine’s reduced presence after electrolysis points to dynamic changes in the surface composition, potentially enhancing the accessibility of active sites. These findings confirm the multifunctional nature of the CuBr-(P(SFc)_3_ composites, where the interplay between copper centers and the trithiophosphite ligand framework enables effective CO_2_ reduction. The stability of the ligand and the tunability of copper’s oxidation state are critical factors that contribute to the composite’s catalytic efficiency and durability.

The selective formation of methanol by composite **1** can be attributed to a catalytic surface that evolves under electrochemical conditions to favor the formation of isolated, mononuclear active sites. These sites are electronically competent for the multi-electron reduction of a single CO_2_ molecule but are sterically and spatially incapable of facilitating the critical C-C coupling step. A crucial piece of evidence for the nature of the active sites in the 1:1 composite comes from the post-electrolysis powder X-ray diffraction analysis. The PXRD pattern for composite **1** after 5 h of catalysis shows a complete disappearance of the characteristic diffraction peaks associated with the crystalline CuBr phase. While the peaks corresponding to the P(SFc)_3_ ligand are retained, the overall pattern is dominated by a broad, amorphous background. This loss of CuBr crystallinity is not indicative of catalyst degradation or leaching, as the system maintains excellent stability over the electrolysis period. Instead, it signifies a profound structural transformation of the pre-catalyst. The CuBr is consumed and redistributed into a new, catalytically active phase that is X-ray amorphous. This suggests the formation of discrete, molecular Cu^+^ ligand complexes or highly dispersed nanoclusters that are too small or disordered to coherently diffract X-rays. In this scenario, the P(SFc)_3_ ligand coordinates to the Cu^+^ ions, effectively dispersing them throughout the carbon paste matrix and preventing their aggregation into larger, crystalline domains. The pre-catalyst thus serves as a precursor to a more dynamic and molecularly defined catalytic system.

In stark contrast to the 1:1 system, the 2:1 composite **2** provides the necessary structural and electronic environment to overcome the kinetic barrier for C-C bond formation, thereby opening the reaction pathway to ethanol. The excess copper in the formulation is not a passive component but the key factor that drives the self-assembly of a distinct active site architecture characterized by a high density of proximal, cooperatively acting Cu^+^ centers.

The formation of C_2+_ products like ethanol is widely accepted to proceed via a rate-determining C-C coupling step, most commonly the dimerization of two adsorbed *CO intermediates on adjacent active sites (*CO + *CO → *OCCO) [24]. This step has a significant activation energy barrier, and overcoming it requires a specific catalyst architecture that can facilitate the interaction of the two intermediates and stabilize the coupling transition state.

The high local density of proximal Cu^+^ sites within the copper-rich domains of composite **2** directly addresses this primary mechanistic hurdle. When CO molecules adsorb on adjacent Cu^+^ sites, the spatial barrier for their interaction is minimized, dramatically increasing the probability of a successful bimolecular coupling event. The ligand framework, by holding these Cu^+^ centers together, effectively creates a supramolecular template for C-C bond formation. Extensive research has shown that the interface between different copper species, particularly adjacent Cu^+^ and Cu^0^ sites, is highly effective at lowering the activation energy for C-C coupling [8]. The XPS data confirms that composite **2** is particularly rich in the crucial Cu^+^ species, which is known to facilitate the initial adsorption and stabilization of *CO intermediates. Once the C-C bond is formed, subsequent hydrogenation steps proceed on the multinuclear site to yield ethanol. In the 2:1 composite, the ligand acts as a structural template. Beyond stabilizing the electronic states of the copper centers, it defines the spatial arrangement between them. This transforms the system from a collection of isolated sites into a cooperative ensemble, where adjacent centers function synergistically to facilitate complex chemical transformationsTo place these observations in a broader context, Table 1 summarizes representative Cu-based CO_2_RR systems reported for alcohol formation and compares their key operating conditions, dominant products, selectivity metrics, and stability with the present CuBr-P(SFc)_3_ composites. The comparison highlights that the highest reported Faradaic efficiencies for ethanol or methanol are typically achieved using carefully engineered Cu nanostructures, Cu(0)/Cu(I) interfaces, or high-density/adjacent Cu site architectures. In contrast, the distinctive feature of our approach lies in the stoichiometry-controlled tuning of alcohol selectivity within the same hybrid Cu(I)–ligand platform: under identical electrode design and electrolyte conditions, changing the CuBr:P(SFc)_3_ ratio shifts the product distribution from predominantly methanol (1:1) to ethanol (2:1).

## 3. Materials and Methods

### 3.1. Electrochemistry

Linear sweep voltammograms were recorded using a BASi Epsilon Eclipse potentiostat (West Lafayette, IN, USA). The device includes a measuring unit, a DellOptiplex 320 personal computer with Epsilon-EC-USB-V200 software (Version 3.0.79.1). 0.1 M potassium bicarbonate was used as a supporting electrolyte. A glassy carbon electrode modified with carbon paste (surface area 1 mm^2^) served as the working electrode. Ag/AgCl (0.01 M KCl) was used as a reference electrode. A platinum wire was used as an auxiliary electrode. The scanning rate was 100 mV s^−1^. Measurements were carried out in a thermostatted electrochemical cell (5 mL) under a nitrogen atmosphere. Between measurements or before recording the voltammetric wave, the aqueous solution was actively stirred with a magnetic stirrer in an atmosphere of constant inert gas flow, which was passed through a drying system and then through a nickel-based cleaning system BI-GAS cleaner (manufactured by Modern Laboratory Equipment LLC, Novosibirsk, Russia) to remove trace amounts of oxygen.

Carbon paste electrodes were prepared using a phosphonium-based ionic liquid as a conductive binder, following our previously reported approach for RTIL-CPEs. The graphite powder was thoroughly mixed with tri(tert-butyl)(dodecyl)phosphonium tetrafluoroborate to obtain a homogeneous base paste with a graphite-to-ionic liquid weight ratio of 90:10 (*w*/*w*).

To prepare modified electrodes, 30 wt% of the base paste was replaced with the investigated component(s). Thus, the final composition of the working pastes was: 63 wt% graphite, 7 wt% ionic liquid, and 30 wt% additive. As a practical example, for a 100 mg batch of paste this corresponds to 63 mg graphite, 7 mg ionic liquid, and 30 mg of the investigated material.

For composite **1**, the 30 wt% additive was the CuBr–P(SFc)_3_ mixture prepared with a 1:1 molar ratio of CuBr to P(SFc)_3_. For composite **2**, the additive was the CuBr–P(SFc)_3_ mixture prepared with a 2:1 molar ratio of CuBr to P(SFc)_3_. Control pastes containing only CuBr or only P(SFc)_3_ were prepared analogously by using the corresponding component as the 30 wt% additive. The resulting pastes were applied to the glassy carbon electrode surface and gently compacted/smoothed before electrochemical measurements.

The phosphonium-based ionic liquid was employed as a conductive binder in the carbon paste electrode to ensure homogeneous dispersion of the copper–ligand composite within the graphite matrix and to provide stable and reproducible electrochemical performance. Phosphonium RTIL-based CPEs are known to exhibit high conductivity, excellent stability, and an unusually wide electrochemical window, clearly outperforming traditional paraffin-based CPEs that suffer from high resistance and variable composition.

Because the ionic liquid content was kept constant for all electrodes, the differences in CO_2_RR selectivity between the 1:1 and 2:1 CuBr-to-ligand composites reflect the effect of copper loading and local site proximity rather than changes in the binder environment.

### 3.2. Preparative Electrolysis

All reactions were performed under a dry argon atmosphere to prevent contamination by oxygen or moisture. Preparative electrolysis was conducted using a B5-49 power supply in a 40 mL three-electrode cell with separate anodic and cathodic compartments. The working electrode potential was measured relative to an Ag/AgCl (0.01 M NaCl) reference electrode using a B7-27 DC voltmeter. The reference electrode consisted of two compartments separated by a Vicor membrane, with the second compartment containing a saturated aqueous solution of the background electrolyte. The working electrode was a U-shaped glassy carbon paste electrode (GC-CPE) with a surface area of 48.00 cm^2^. A ceramic membrane with a pore size of 3 μm was employed to separate the compartments. Throughout the electrolysis process, the electrolyte was continuously stirred using a magnetic stirrer, while an inert argon gas stream, purified to remove oxygen and other impurities, was introduced into the system. The cathode was a glassy carbon electrode modified with carbon paste containing coordination polymers, and the catholyte consisted of a saturated KHCO_3_ solution as the background electrolyte.

### 3.3. Faradaic Efficiency (Current Efficiency) Calculations

Faradaic efficiencies (FE, also referred to as current efficiencies, CE) for liquid products were calculated from the experimentally measured total charge passed during potentiostatic electrolysis. The total charge was obtained by integration of the current–time curve:

$$Q_{total}=\;\int_{0}^{t}I\left(t\right)dt$$

The amount of methanol/ethanol in the catholyte was determined by GC-FID using external calibration (peak area integration and calibration curves prepared in the same electrolyte matrix). The FE for product i was calculated as:

$${FE}_{i}\left(\%\right)=\;\frac{n_{i}FN_{i}}{Q_{total}}\times 100$$

where *F* = 96,485 C mol^−1^, *N_i_* is the number of moles of product *i*, and *n_i_* is the number of electrons required per molecule: *n* = 6 for methanol and *n* = 12 for ethanol. The reported *FE* values were calculated using *Q_total_* = 50 C.

### 3.4. Gas Chromatographic Analysis of Liquid Products

Liquid-phase products formed during CO_2_ electroreduction were analyzed using an Agilent Technologies 6890N (Santa Clara, CA, USA) gas chromatograph equipped with a flame ionization detector (FID). Separation was carried out on a PEG capillary column (50 m). Methanol and ethanol were identified by comparing their retention times with those of authentic standards analyzed under identical conditions. Quantification was performed by integration of the corresponding peak areas and external calibration using standard solutions prepared in the same electrolyte matrix.

### 3.5. SEM

The surfaces of the electrodes were characterized using a Carl Zeiss Evo LS10 scanning electron microscope. Sample images were obtained using a secondary electron detector. Elemental analysis of the samples was performed using an INCA X-Max energy dispersive spectrometer. The samples were studied at the probe current IProbe = 150 pA, at the accelerating voltage EHT = 20 kV.

### 3.6. Powder XRD

Powder X-ray diffraction (PXRD) patterns were recorded using an X-ray powder diffractometer (DX-2700BH, Haoyuan, Dandong, China), with Cu radiation, Kα = 1.5418 Å, at 40 kV and 30 mA. Room-temperature data were collected in the reflection mode with a flat-plate sample. Samples were placed on the surface of a standard zero-diffraction silicon plate, which reduces background scattering. Patterns were recorded in the 2θ range between 3° and 70° in 0.02° steps with a step time of 0.5 s. For the samples several diffractograms were obtained, which were summed. Processing of the obtained data was performed using DIFFRAC.EVA software (version 4.2.1, Bruker AXS, Karlsruhe, Germany) [27] software packages.

### 3.7. XPS

An X-ray photoelectron spectroscopy chamber was equipped with the Mg-K_α_ X-ray source operating at 12.5 kV and 250 W, along with the Phoibos150 hemispherical energy analyzer of photoelectrons (all from SPECS GmbH, Berlin, Germany). The survey XPS spectra were recorded in the range of 0–1000 eV with an energy step of 0.5 eV and a pass energy of 100 eV. High-resolution spectra were recorded in limited energy ranges of interest by averaging over 20 scans for every element with an energy step of 0.1 eV and a pass energy of 40 eV. The collected XPS spectra were analyzed using CasaXPS software (version 2.3.25, Casa Software Ltd., Teignmouth, UK) [28]. The energy scale for all spectra was calibrated using the C 1s (C–C, C–H) peak with a known value of 284.8 eV [22]. The background from the spectra was subtracted using a Shirley-type background.

## 4. Conclusions

In this work, novel trisferrocenyltrithiophosphite-copper(I) bromide composites with Cu to ligand molar ratios of 1:1 and 2:1 were synthesized and evaluated as catalysts for CO_2_ electroreduction. Comprehensive characterization using PXRD, SEM, XPS, and electrochemical techniques confirmed the structural integrity, stability, and activity of the composites.

The 1:1 composite favored methanol production, while the 2:1 composite shifted the distribution toward ethanol (with FEs of 5.79% and 9.26%, respectively). This demonstrates that the Cu:ligand ratio provides a simple handle to tune alcohol selectivity. Although Faradaic efficiencies are currently limited by competitive pathways, these results showcase the potential of Cu–trisferrocenyltrithiophosphite composites as stable and tunable electrocatalysts. PXRD confirmed the preservation of structural features after electrocatalysis, while SEM and elemental mapping revealed the formation of active catalytic centers. XPS analysis indicated changes in the oxidation states of copper, with Cu(I) being the dominant active species during catalysis. Potentiostatic testing further verified the stability of the composites, showing minimal current density decline over extended electrolysis.

Future efforts will focus on optimizing the ligand environment and exploring additional Cu-ligand systems to further enhance the efficiency and selectivity of CO_2_ reduction.

## Figures and Tables

**Table 1:** Comparison of representative Cu-based electrocatalysts for CO_2_RR to alcohols with the present work.

Catalyst (Type)	Key Conditions (As Reported)	Potential	Major Alcohol(s)	Selectivity (FE)	Ref.
Activated Cu mesh (nanodendritic Cu after multistep activation)	KHCO_3_	−1.0 V vs. RHE	EtOH	13%	[19]
Cu@Cu_2_O-400 °C	0.5 M KHCO_3_	−0.7 V vs. RHE	MeOH	45%	[25]
Cu single-atom-derived dynamic clusters	Carbon-supported; operando reconstruction	−0.7 V vs. RHE	EtOH	91%	[26]
Adjacent Cu single atoms (high-density Cu-N_3_ motifs)	H-type cell	−1.1 V vs. RHE	EtOH	81.9%	[14]
CuBr–P(SFc)_3_ composites in RTIL-CPE	0.1 M KHCO_3_; identical CPE architecture	−0.8, −1.2 V vs. Ag/AgCl	MeOH (1:1); EtOH (2:1)	5.8% (MeOH); 9.3% (EtOH)	**this work**

## Data Availability

Data are contained within the article.

## References

[1] Yu J., Wang J., Ma Y., Zhou J., Wang Y., Lu P., Yin J., Ye R., Zhu Z., Fan Z. (2021). Recent Progresses in Electrochemical Carbon Dioxide Reduction on Copper-Based Catalysts toward Multicarbon Products. Adv. Funct. Mater..

[2] Zhu Y., Yang X., Peng C., Priest C., Mei Y., Wu G. (2021). Carbon-Supported Single Metal Site Catalysts for Electrochemical CO_2_ Reduction to CO and Beyond. Small.

[3] Li Y., Dong Z., Jiao L. (2019). Multifunctional Transition Metal-Based Phosphides in Energy-Related Electrocatalysis. Adv. Energy Mater..

[4] Chakraborty B., Menezes P.W., Driess M. (2020). Beyond CO_2_ Reduction: Vistas on Electrochemical Reduction of Heavy Non-metal Oxides with Very Strong E—O Bonds (E = Si, P, S). J. Am. Chem. Soc..

[5] Zhai R., Zhang L., Gu M., Zhao X., Zhang B., Cheng Y., Zhang J. (2023). A Review of Phosphorus Structures as CO_2_ Reduction Photocatalysts. Small.

[6] Khrizanforova V., Shekurov R., Miluykov V., Khrizanforov M., Bon V., Kaskel S., Gubaidullin A., Sinyashin O., Budnikova Y. (2020). 3D Ni and Co redox-active metal–organic frameworks based on ferrocenyl diphosphinate and 4,4′-bipyridine ligands as efficient electrocatalysts for the hydrogen evolution reaction. Dalton Trans..

[7] Shekurov R., Khrizanforova V., Gilmanova L., Khrizanforov M., Miluykov V., Kataeva O., Yamaleeva Z., Burganov T., Gerasimova T., Khamatgalimov A. (2019). Zn and Co redox active coordination polymers as efficient electrocatalysts. Dalton Trans..

[8] Khrizanforova V.V., Musina E.I., Khrizanforov M.N., Gerasimova T.P., Katsyuba S.A., Spiridonova Y.S., Islamov D.R., Kataeva O.N., Karasik A.A., Sinyashin O.G. (2015). Unexpected ligand effect on the catalytic reaction rate acceleration for hydrogen production using biomimetic nickel electrocatalysts with 1,5-diaza-3,7-diphosphacyclooctanes. J. Organomet. Chem..

[9] Khrizanforova V.V., Morozov V.I., Strelnik A.G., Spiridonova Y.S., Khrizanforov M.N., Burganov T.I., Katsyuba S.A., Latypov S.K., Kadirov M.K., Karasik A.A. (2017). In situ electrochemical synthesis of Ni(I) complexes with aminomethylphosphines as intermediates for hydrogen evolution. Electrochim. Acta.

[10] Song W., Xiao C., Ding J., Huang Z., Yang X., Zhang T., Mitlin D., Hu W. (2023). Review of Carbon Support Coordination Environments for Single Metal Atom Electrocatalysts (SACS). Adv. Mater..

[11] Hua Y., Zhu C., Zhang L., Dong F. (2024). Designing Surface and Interface Structures of Copper-Based Catalysts for Enhanced Electrochemical Reduction of CO_2_ to Alcohols. Materials.

[12] Scott N.W.J., Ford M.J., Jeddi N., Eyles A., Simon L., Whitwood A.C., Tanner T., Willans C.E., Fairlamb I.J.S. (2021). A Dichotomy in Cross-Coupling Site Selectivity in a Dihalogenated Heteroarene: Influence of Mononuclear Pd, Pd Clusters, and Pd Nanoparticles—The Case for Exploiting Pd Catalyst Speciation. J. Am. Chem. Soc..

[13] Karapinar D., Huan N.T., Ranjbar Sahraie N., Li J., Wakerley D., Touati N., Zanna S., Taverna D., Galvão Tizei L.H., Zitolo A. (2019). Electroreduction of CO_2_ on Single-Site Copper-Nitrogen-Doped Carbon Material: Selective Formation of Ethanol and Reversible Restructuration of the Metal Sites. Angew. Chem. Int. Ed..

[14] Xia W., Xie Y., Jia S., Han S., Qi R., Chen T., Xing X., Yao T., Zhou D., Dong X. (2023). Adjacent Copper Single Atoms Promote C–C Coupling in Electrochemical CO_2_ Reduction for the Efficient Conversion of Ethanol. J. Am. Chem. Soc..

[15] Zhu J., Luo L., Lai C., He B., Chen P. (2025). Confining bismuth in oxygen-defective BiVO_4_ through chemical reconstruction engineering for enhanced electrocatalytic formate generation. Chem. Eng. J..

[16] Zhu J., Zhou G., Tong Y., Chen L., Chen P. (2024). Vanadium Oxide Clusters Mediated Bismuth-Tin Alloy for Accelerated Dynamics of Electrocatalytic CO_2_ Conversion. Adv. Funct. Mater..

[17] Zhang B., Jiao H., Michalik D., Kloß S., Deter L.M., Selent D., Spannenberg A., Franke R., Börner A. (2016). Hydrolysis Stability of Bidentate Phosphites Utilized as Modifying Ligands in the Rh-Catalyzed n-Regioselective Hydroformylation of Olefins. ACS Catal..

[18] Wu Y., Jiang Z., Lu X., Liang Y., Wang H. (2019). Domino electroreduction of CO_2_ to methanol on a molecular catalyst. Nature.

[19] Rahaman M., Dutta A., Zanetti A., Broekmann P. (2017). Electrochemical Reduction of CO_2_ into Multicarbon Alcohols on Activated Cu Mesh Catalysts: An Identical Location (IL) Study. ACS Catal..

[20] Shekurov R., Khrizanforov M., Gerasimova T., Yamaleeva Z., Ivshin K., Lakomkina A., Bezkishko I., Kononov A., Sinyashin O., Budnikova Y. (2020). Electrochemical Properties and Structure of Multi-Ferrocenyl Phosphorus Thioesters. Molecules.

[21] Khrizanforov M.N., Arkhipova D.M., Shekurov R.P., Gerasimova T.P., Ermolaev V.V., Islamov D.R., Miluykov V.A., Kataeva O.N., Khrizanforova V.V., Sinyashin O.G. (2015). Novel paste electrodes based on phosphonium salt room temperature ionic liquids for studying the redox properties of insoluble compounds. J. Solid State Electrochem..

[22] Wu H., Yu H., Chow Y.L., Webley P.A., Zhang J. (2024). Toward durable CO_2_ electroreduction with Cu-based catalysts via understanding their deactivation modes. Adv. Mater..

[23] Biesinger M.C. (2017). Advanced analysis of copper X-ray photoelectron spectra. Surf. Interface Anal..

[24] Schouten K.J.P., Kwon Y., van der Ham C.J.M., Qin Z., Koper M.T.M. (2011). A new mechanism for the selectivity to C1 and C2 species in the electrochemical reduction of carbon dioxide on copper electrodes. Chem. Sci..

[25] Yang X., Cheng J., Yang X., Xu Y., Sun W., Zhou J. (2022). MOF-derived Cu@Cu_2_O heterogeneous electrocatalyst with moderate intermediates adsorption for highly selective reduction of CO_2_ to methanol. Chem. Eng. J..

[26] Xu H., Rebollar D., He H., Chong L., Liu Y., Liu C., Sun C.-J., Li T., Muntean J.V., Winans R.E. (2020). Highly selective electrocatalytic CO_2_ reduction to ethanol by metallic clusters dynamically formed from atomically dispersed copper. Nat. Energy.

[27] Bruker AXS (2005). DIFFRAC Plus Evaluation Package EVA, Version 11; User’s Manual.

[28] Fairley N., Fernandez V., Richard-Plouet M., Guillot J., Walton J., Dietrich P., Menneken M., Stromberg B., Greiner S. (2021). Systematic and automated approach to analyzing X-ray photoelectron spectra using CasaXPS. Appl. Surf. Sci. Adv..

